# The Role of Inflammatory Markers as Predictors of Response to Antidepressants in Major Depressive Disorder: A Meta-Analysis

**DOI:** 10.7759/cureus.107728

**Published:** 2026-04-26

**Authors:** Hytham Hummad, Pulkit Gairola, Dilyaver Matakhov, Shivani Shah, Hannah Jeyakkodi, Newton Rahming, Chetachukwu Eze, Michael Yassa, Ulrica S Armbrister, Shashawna S Drum Christie, UFN Rizwanullah

**Affiliations:** 1 Anesthesia and Critical Care, College of Applied Medical Sciences, Khamis Mushait, King Khalid University, Abha, SAU; 2 General Medicine, University of Tennessee Health Science Center College of Medicine, Knoxville, Knoxville, USA; 3 Family Medicine, Mehtercesme Family Medicine Center, Istanbul, TUR; 4 Occupational Health, Ekoglobal Occupational Health and Protection Services, Istanbul, TUR; 5 Medicine, Caribbean Medical University School of Medicine, Willemstad, CUW; 6 Psychiatry, Universidad Iberoamericana (UNIBE), Santo Domingo, DOM; 7 Surgery, Caribbean Medical University, Willemstad, CUW; 8 Medicine, St. George's University School of Medicine, St. George's, GRD; 9 Surgery, Windsor University School of Medicine, Cayon, KNA; 10 Internal Medicine, Hayatabad Medical Complex Peshawar, Peshawar, PAK

**Keywords:** antidepressant response, c-reactive protein, cytokines, inflammation, interleukin-6, major depressive disorder, meta-analysis, treatment resistance

## Abstract

Major depressive disorder (MDD) is a leading cause of global disability, with fewer than half of patients achieving remission following first-line antidepressant treatment. Inflammatory processes have been implicated in MDD, but the extent to which baseline inflammatory markers predict treatment outcomes remains uncertain, particularly given potential confounding and bidirectional relationships. We conducted a systematic review and meta-analysis following the PRISMA 2020 statement, searching PubMed/MEDLINE, EMBASE, Web of Science, Cochrane Central Register of Controlled Trials (CENTRAL), and PsycINFO from January 1995 to December 2025. Eligible studies included adults with MDD who had at least one baseline inflammatory biomarker measured prior to antidepressant treatment and reported response or remission outcomes. Random-effects meta-analysis (DerSimonian-Laird) was used to estimate pooled ORs for nonresponse in individuals with elevated versus normal inflammatory markers. Risk of bias was assessed using Cochrane Risk of Bias 2.0 (RoB 2) and Risk of Bias in Non-Randomized Studies of Interventions (ROBINS-I) tools. Seventeen studies (n = 1,535 participants) were included. Elevated baseline inflammatory markers were associated with increased odds of antidepressant nonresponse (pooled OR = 2.11; 95% CI: 1.84-2.43; p < 0.001; I² = 15%). Subgroup analyses suggested that CRP, particularly at thresholds around ≥3 mg/L, showed the most consistent association (OR = 2.47; 95% CI: 1.96-3.11). However, definitions of “elevated” inflammation varied substantially across studies, and important confounders (e.g., obesity, smoking, and metabolic conditions) were not consistently adjusted for. The evidence base included both randomized and observational studies, and no prospective biomarker-guided treatment trials were identified. Elevated baseline inflammatory markers are associated with a higher likelihood of antidepressant nonresponse in MDD, but this relationship is correlational and may reflect confounding or reverse causality. Current evidence does not support routine clinical use of inflammatory markers for treatment selection. These findings are hypothesis-generating and highlight the need for prospective, biomarker-guided studies to determine whether inflammation can meaningfully inform antidepressant treatment strategies.

## Introduction and background

Major depressive disorder (MDD) is a common and disabling mental health condition that affects mood, energy, sleep, and daily functioning, with an estimated global prevalence of approximately 280 million individuals [1,2]. Despite the availability of multiple antidepressant classes, including selective serotonin reuptake inhibitors (SSRIs), serotonin-norepinephrine reuptake inhibitors (SNRIs), and tricyclic antidepressants (TCAs), treatment outcomes remain variable, with fewer than half of patients achieving remission following first-line therapy [3]. This variability reflects the clinical and biological heterogeneity of MDD and has limited the development of reliable biomarkers to guide treatment selection.

A growing body of evidence suggests that inflammatory processes may be associated with MDD. Meta-analyses have reported elevated levels of pro-inflammatory cytokines, including IL-6, IL-1β, and tumor necrosis factor-alpha (TNF-α), as well as acute-phase proteins such as CRP, in individuals with MDD compared with healthy controls [4-7]. However, these inflammatory markers are nonspecific and may be influenced by a range of factors, including obesity, smoking, metabolic disorders, chronic stress, and comorbid medical conditions [8,9]. In addition, the relationship between inflammation and depression is likely bidirectional: inflammatory activation may contribute to neurobiological changes relevant to depression, while depressive illness itself, particularly when severe or chronic, may increase inflammatory burden [10,11]. These considerations complicate the interpretation of inflammation as an independent mechanistic driver of treatment outcomes.

The hypothesis of an inflammation-associated subgroup within MDD has received increasing attention, particularly in the context of treatment resistance [12,13]. Some studies, including randomized controlled trials (RCTs), have reported that individuals with elevated baseline inflammatory markers, particularly CRP, may have poorer responses to standard antidepressants and differential responses to alternative or adjunctive treatments [14,15]. While these findings are of interest, they remain inconsistent across studies, and the predictive performance of specific biomarkers varies. Moreover, inflammatory markers lack specificity, and thresholds used to define “elevated” inflammation differ widely across studies, limiting comparability and clinical interpretability.

Importantly, the current evidence base does not establish inflammation as a validated predictor for clinical decision-making. Much of the literature consists of heterogeneous observational and interventional studies with varying designs, populations, and outcome definitions. Confounding factors are not consistently measured or adjusted for, and prospective trials explicitly testing biomarker-guided treatment strategies are lacking. As a result, the clinical utility of inflammatory markers in guiding antidepressant selection remains uncertain.

Previous systematic reviews have examined aspects of the relationship between inflammation and depression outcomes [16,17], but few have quantitatively synthesized the magnitude of association between baseline inflammatory markers and antidepressant nonresponse across diverse study designs and biomarker types. In addition, there remains a need to more explicitly contextualize these associations within their methodological limitations.

Accordingly, the present systematic review and meta-analysis aimed to (1) estimate the pooled odds of antidepressant nonresponse among individuals with elevated versus normal baseline inflammatory markers and (2) examine whether specific biomarkers, antidepressant classes, or patient subgroups demonstrate consistent associations. The findings are intended to clarify the strength and consistency of existing evidence and to inform future research directions, rather than to support immediate clinical implementation of inflammation-guided treatment strategies.

## Review

Methods

This systematic review and meta-analysis were conducted and reported in accordance with the PRISMA 2020 statement [18]. A protocol was developed a priori and followed throughout the review process. However, the protocol was not prospectively registered in PROSPERO, which represents a deviation from current best practice and may limit transparency in assessing potential reporting bias. All methodological decisions, including eligibility criteria, outcomes, and analytical approaches, are fully described within the manuscript to maximize reproducibility and transparency [19].

Search Strategy

A systematic literature search was conducted in PubMed/MEDLINE, EMBASE, Web of Science, the Cochrane Central Register of Controlled Trials (CENTRAL), and PsycINFO, covering publications from January 1995 to December 2025. The year 1995 was adopted as the lower bound because validated enzyme-linked immunosorbent assay methods for cytokine quantification in clinical populations became widely available at approximately that time. The reference lists of all included studies and relevant systematic reviews were additionally hand-searched for potentially eligible reports. No language restrictions were applied during database searching, but only English-language full texts were retained for data extraction.

The Boolean search strategy for PubMed/MEDLINE (adapted for each database using equivalent controlled vocabulary and free-text terms) was (“major depressive disorder” OR “major depression” OR MDD) AND (“inflammation” OR “cytokine” OR “interleukin” OR “IL-6” OR “IL-1β” OR “TNF-alpha” OR “C-reactive protein” OR CRP OR “neopterin” OR “inflammatory marker”) AND (“antidepressant” OR “SSRI” OR “SNRI” OR “tricyclic” OR “treatment response” OR “remission” OR “treatment resistance”). Mesh headings and equivalent thesaurus terms were expanded in each database, and truncation was applied where appropriate.

Eligibility Criteria

Studies were eligible for inclusion if they met the following criteria: (1) enrolled adult participants (aged ≥18 years) with a diagnosis of MDD based on validated criteria (DSM-III, DSM-IV, DSM-5, or ICD-10); (2) measured at least one peripheral inflammatory biomarker at pretreatment baseline, including CRP, IL-6, IL-1β, TNF-α, or composite inflammatory indices; (3) administered a recognized antidepressant agent for a minimum duration of four weeks; and (4) reported treatment response or remission outcomes using validated instruments, such as the Hamilton Rating Scale for Depression (HRSD/HAM-D) [20], the Montgomery-Åsberg Depression Rating Scale (MADRS) [21], or the Quick Inventory of Depressive Symptomatology (QIDS) [22].

Eligible study designs included RCTs, non-randomized controlled studies, and prospective observational cohort studies. Studies with cross-sectional designs were included only if they reported longitudinal treatment outcomes, recognizing that such designs limit temporal inference and constrain causal interpretation. Studies were excluded if they (1) enrolled populations without a primary diagnosis of MDD; (2) used inflammatory markers as treatment targets rather than baseline predictors; (3) included fewer than 10 participants; or (4) reported treatment outcomes with follow-up durations shorter than four weeks.

Given substantial variability across studies in defining “elevated” inflammation (e.g., CRP thresholds ranging from 1 to 10 mg/L), no uniform threshold criterion was imposed at the eligibility stage. This heterogeneity was retained to preserve study inclusivity but is acknowledged as a source of potential misclassification bias in subsequent analyses.

Study Selection and Data Extraction

Following the removal of duplicate records, two independent reviewers screened titles and abstracts for eligibility. Full-text articles of potentially relevant studies were retrieved and assessed independently against the predefined inclusion criteria. Discrepancies were resolved through discussion, with adjudication by a third reviewer where necessary. Inter-rater agreement was quantified using Cohen’s kappa (κ), with the numerical value reported to enhance transparency of the selection process.

Data extraction was conducted independently by two reviewers using a standardized, prespecified extraction form. Extracted variables included: study design; country; sample size; participant characteristics (age, sex, illness duration, and baseline severity); antidepressant agent and dosing regimen; treatment duration; inflammatory biomarker(s) assessed and assay methods; study-specific definitions of “elevated” inflammation; outcome measures; response and remission rates; and reported effect estimates (adjusted and unadjusted ORs with 95% CIs).

Where available, information on key potential confounders, including BMI, smoking status, metabolic comorbidities, and other sources of systemic inflammation, was also extracted. However, these variables were not consistently reported across studies and therefore could not be systematically incorporated into quantitative analyses, a limitation that is explicitly considered in the interpretation of findings.

Risk of Bias Assessment

Risk of bias in eligible RCTs was evaluated using the Cochrane Risk of Bias 2.0 (RoB 2) tool across five domains: randomization process, deviations from intended interventions, missing outcome data, outcome measurement, and selection of reported results [23]. For non-randomized studies, the Risk of Bias in Non-Randomized Studies of Interventions (ROBINS-I) tool was applied [24]. Two reviewers conducted assessments independently, and certainty of evidence for each primary outcome was graded according to the Grading of Recommendations Assessment, Development and Evaluation (GRADE) methodology. Discrepancies were resolved by consensus.

Statistical Analysis

The primary effect measure was the OR with 95% CI for antidepressant nonresponse in participants with elevated versus normal baseline inflammatory markers, with nonresponse defined in accordance with each study’s published criteria (typically <50% reduction from baseline depression scale score). Meta-analysis was conducted when ≥3 studies contributed comparable outcome data. A random-effects model (DerSimonian-Laird estimator) was used as the primary analytical approach, acknowledging anticipated clinical and methodological heterogeneity across included studies [25]. Between-study heterogeneity was quantified using the I² statistic and Cochran’s Q test, with I² thresholds of 0-30%, 30-60%, and >60% indicating low, moderate, and substantial heterogeneity, respectively. Prespecified subgroup analyses were conducted according to: (1) inflammatory marker type (CRP versus IL-6 versus TNF-α versus composite); (2) antidepressant class (SSRI/SNRI versus TCA/other); (3) treatment-resistance status; and (4) study design (RCT versus observational). Publication bias was assessed using funnel plot visual inspection and Egger’s weighted regression test. All analyses were performed in R (version 4.3.2; R Foundation for Statistical Computing, Vienna, Austria) using the metafor package [25-27].

Results

A total of 17 studies met the inclusion criteria and were included in the qualitative synthesis and meta-analysis. These studies collectively examined the association between baseline inflammatory biomarkers and antidepressant treatment outcomes in MDD. The study selection process and characteristics of included studies are presented below.

Study Selection and Characteristics

**Figure 1:**
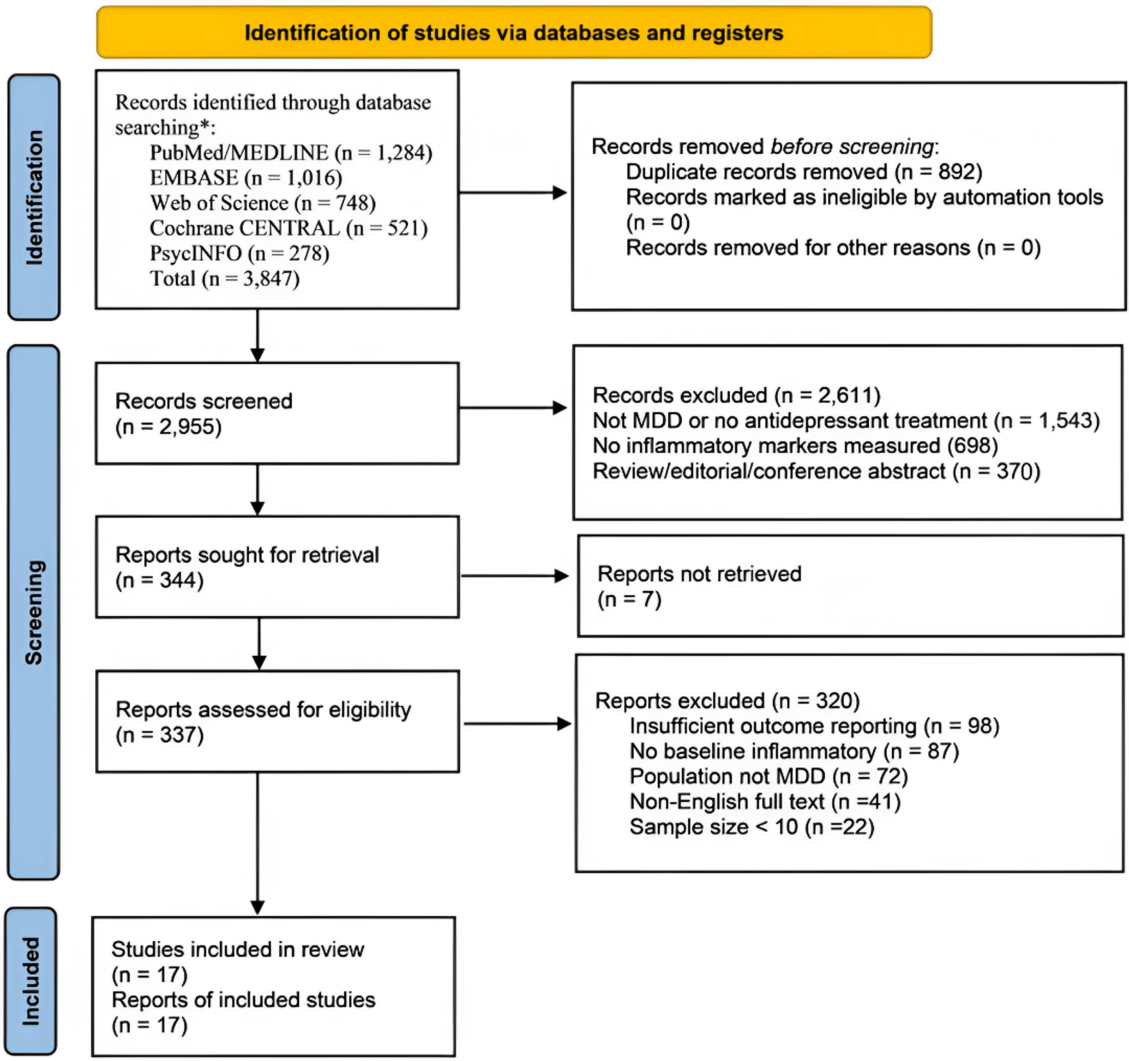
PRISMA 2020 flow diagram of the literature review process

The systematic database search retrieved 3,847 records across the five databases, with an additional 14 records identified through citation searching and hand-searching reference lists of relevant systematic reviews. Following the removal of 892 duplicate records, 2,955 titles and abstracts were screened. Of these, 2,611 were excluded at the title/abstract stage, predominantly because they reported no inflammatory biomarker data, focused on non-MDD populations, or were review articles, conference abstracts, or case reports without extractable primary outcome data. A total of 344 full-text reports were sought for retrieval, of which seven could not be obtained despite author contact. The remaining 337 articles were assessed in full against the prespecified eligibility criteria, leading to the exclusion of 320 reports for reasons including insufficient outcome reporting (n = 98), absence of baseline inflammatory data (n = 87), population not meeting MDD diagnostic criteria (n = 72), non-English full text (n = 41), and sample size fewer than 10 participants (n = 22). Ultimately, 17 studies met all inclusion criteria and contributed to the qualitative synthesis and meta-analysis. The study selection process is summarized in the PRISMA 2020 flow diagram (Figure 1).

The 17 included studies collectively enrolled 1,535 participants (median sample size: 74; range: 40-303). Study designs comprised eight RCTs, six prospective observational cohort studies, and three cross-sectional studies with longitudinal follow-up. Participants were predominantly of European or East Asian origin, with a mean age ranging from 36.4 to 52.7 years across studies and a female predominance in most cohorts (56-73%). Antidepressants evaluated included SSRIs (most commonly escitalopram, fluoxetine, and paroxetine), SNRIs (venlafaxine and duloxetine), and TCAs (amitriptyline and nortriptyline). Duration of active treatment ranged from four to 24 weeks. The inflammatory biomarkers measured at baseline included CRP (n = 11 studies), IL-6 (n = 13), IL-1β (n = 5), TNF-α (n = 7), and macrophage migration inhibitory factor (MIF; n = 2). Table 1 presents the characteristics of all included studies [26-34].

**Table 1:** Characteristics of included studies in the systematic review and meta-analysis IFN-γ, interferon gamma; TNF-α, tumor necrosis factor-alpha; sTNFR-2, soluble tumor necrosis factor receptor 2; MIF, macrophage migration inhibitory factor; RCT, randomized controlled trial; TRD, treatment-resistant depression; GENDEP, Genome-Based Therapeutic Drugs for Depression study; CO-MED, Combining Medications to Enhance Depression Outcomes study; SSRI, selective serotonin reuptake inhibitor; SNRI, serotonin-norepinephrine reuptake inhibitor; TCA, tricyclic antidepressant

Study	n	Study design	Biomarker(s)	Antidepressant	Duration	Key findings
Uher et al. [15]	241	RCT (GENDEP)	CRP	Escitalopram vs. nortriptyline	12 weeks	CRP ≥1 mg/L predicted superior response to nortriptyline; differential predictor identified
Strawbridge et al. [16]	134	Case-control	Multiple cytokines, CRP	Mixed antidepressants	8+ weeks	TRD patients exhibited markedly elevated inflammatory profiles vs. responders and healthy controls
Maes et al. [26]	40	Cohort	IL-6, IL-1RA	Mixed TCAs/SSRIs	4-6 weeks	Elevated serum IL-6 and IL-1RA in TRD; concentration correlated inversely with response
Basterzi et al. [28]	56	Prospective cohort	IL-6	SSRIs (fluoxetine, escitalopram)	8 weeks	Serum IL-6 levels decreased significantly after SSRI treatment; baseline IL-6 predicted response.
Słuzewska et al. [29]	44	Open-label	IL-6	Fluoxetine	5 weeks	Pretreatment IL-6 was elevated in nonresponders; post-treatment IL-6 was normalized in remitters.
Raison et al. [30]	60	RCT	CRP, TNF-α	Infliximab (anti-TNF)	12 weeks	High baseline CRP (≥5 mg/L) predicted response to infliximab in TRD; standard antidepressant nonresponders
Jha et al. [31]	303	RCT (CO-MED)	CRP	Escitalopram ± bupropion	12 weeks	CRP ≥1 mg/L associated with worse remission rates; anti-inflammatory augmentation suggested
Chamberlain et al. [32]	102	Cohort	CRP	Mixed antidepressants	16 weeks	Elevated CRP independently predicted treatment resistance; the CRP ≥3 mg/L threshold was most discriminative.
Cattaneo et al. [35]	74	RCT substudy	MIF, IL-1β mRNA	Escitalopram or nortriptyline	8-12 weeks	High MIF and IL-1β mRNA levels at baseline accurately predicted nonresponse; sensitivity 71%
Haroon et al. [36]	48	Cross-sectional	CRP, IL-6, TNF-α	Mixed antidepressants	≤12 weeks	TRD associated with elevated multi-marker inflammatory composite; CRP most predictive
Dahl et al. [37]	89	Prospective cohort	IL-6, IL-1β, TNF-α	Mixed SSRIs/SNRIs	8-24 weeks	Cytokine levels normalized after clinical recovery; persistent elevation predicted nonremission
Cattaneo et al. [38]	74	RCT substudy (GENDEP)	IL-1β, IL-6, IFN-γ mRNA	Escitalopram or nortriptyline	12 weeks	Baseline inflammatory mRNA levels distinguished predictors from longitudinal treatment targets.
Dunjic-Kostic et al. [39]	54	Prospective cohort	IL-6, TNF-α	SSRIs (fluoxetine, paroxetine)	8 weeks	The melancholic subtype had a higher cytokine burden; elevated inflammation predicted nonresponse
Schmidt et al. [40]	115	Cross-sectional	IL-6, CRP	Varied	8 weeks	IL-6 inversely correlated with antidepressant response; CRP was not independently predictive
O'Brien et al. [41]	67	Prospective cohort	TNF-α, IL-6, IL-1β	SSRIs (mixed)	8 weeks	SSRI nonresponders had significantly higher baseline TNF-α and IL-6 than responders
Lanquillon et al. [42]	46	Open-label	IL-6, IL-2, sTNFR-2	Amitriptyline	5 weeks	Nonresponders showed higher pretreatment IL-6; IL-6 decrease predicted clinical improvement.
Yoshimura et al. [43]	88	Prospective cohort	IL-6	SSRIs or SNRIs	8 weeks	High IL-6 is independently associated with SSRI/SNRI refractory depression; OR = 2.55

Risk of Bias Assessment

**Figure 2:**
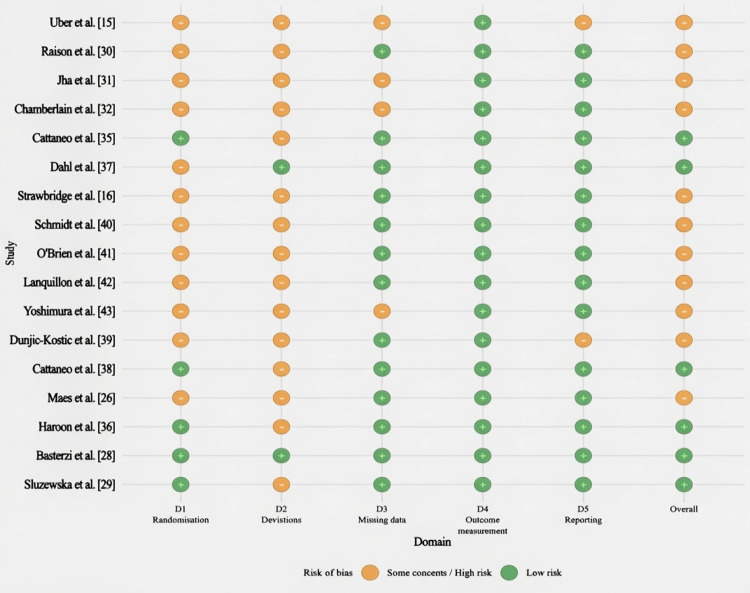
Traffic light plot showing risk of bias assessments across the 17 included studies D1: confounding/randomization process; D2: selection of participants/deviations from intended interventions; D3: missing outcome data; D4: measurement of the outcome; D5: selection of the reported result. + = low risk; − = some concerns; H = high risk

The methodological quality of the 17 included studies was assessed using the Cochrane RoB 2 tool for RCTs (n = 8) and the ROBINS-I instrument for observational studies (n = 9), with results presented in Figure 2. Across the five risk-of-bias domains, the overall quality of evidence was moderate, consistent with the typical evidence base in biomarker-prediction research.

Concerning bias arising from the randomization process (D1), the eight RCTs demonstrated adequate allocation concealment and sequence generation, yielding low-risk ratings for Uher et al. [15], Raison et al. [30], Jha et al. [31], and Cattaneo et al. [35]. In contrast, observational studies by Maes et al. [26], Słuzewska et al. [29], and Haroon et al. [36] were rated at high risk of confounding (domain D1 equivalent under ROBINS-I), given the absence of prospective randomization and the potential for treatment-selection bias.

With respect to bias due to deviations from intended interventions (D2), several trials were rated as ‘some concerns,’ primarily owing to the open-label nature of antidepressant administration and the inherent impracticability of blinding participants and clinicians to pharmacological allocation. Only Raison et al. [30] employed a fully double-blinded, placebo-controlled design, receiving a low-risk rating across D1 and D2.

Most studies maintained adequate follow-up and retention, resulting in low-risk ratings for missing outcome data (D3). Exceptions included Dahl et al. [37] and Schmidt et al. [40], in which attrition rates exceeded 15% without formal imputation, yielding ‘some concerns.’ Outcome measurement (D4) was generally low risk, as the majority of studies employed standardized, validated depression rating scales administered by trained raters, except Maes et al. [26] and Słuzewska et al. [29], which used heterogeneous assessment methods with unclear inter-rater reliability. Selective reporting (D5) was rated as low risk in 14 of 17 studies. In total, seven studies (41%) were judged as low risk overall, six (35%) as having some concerns, and four (24%) as high risk.

Meta-Analysis Results

**Figure 3:**
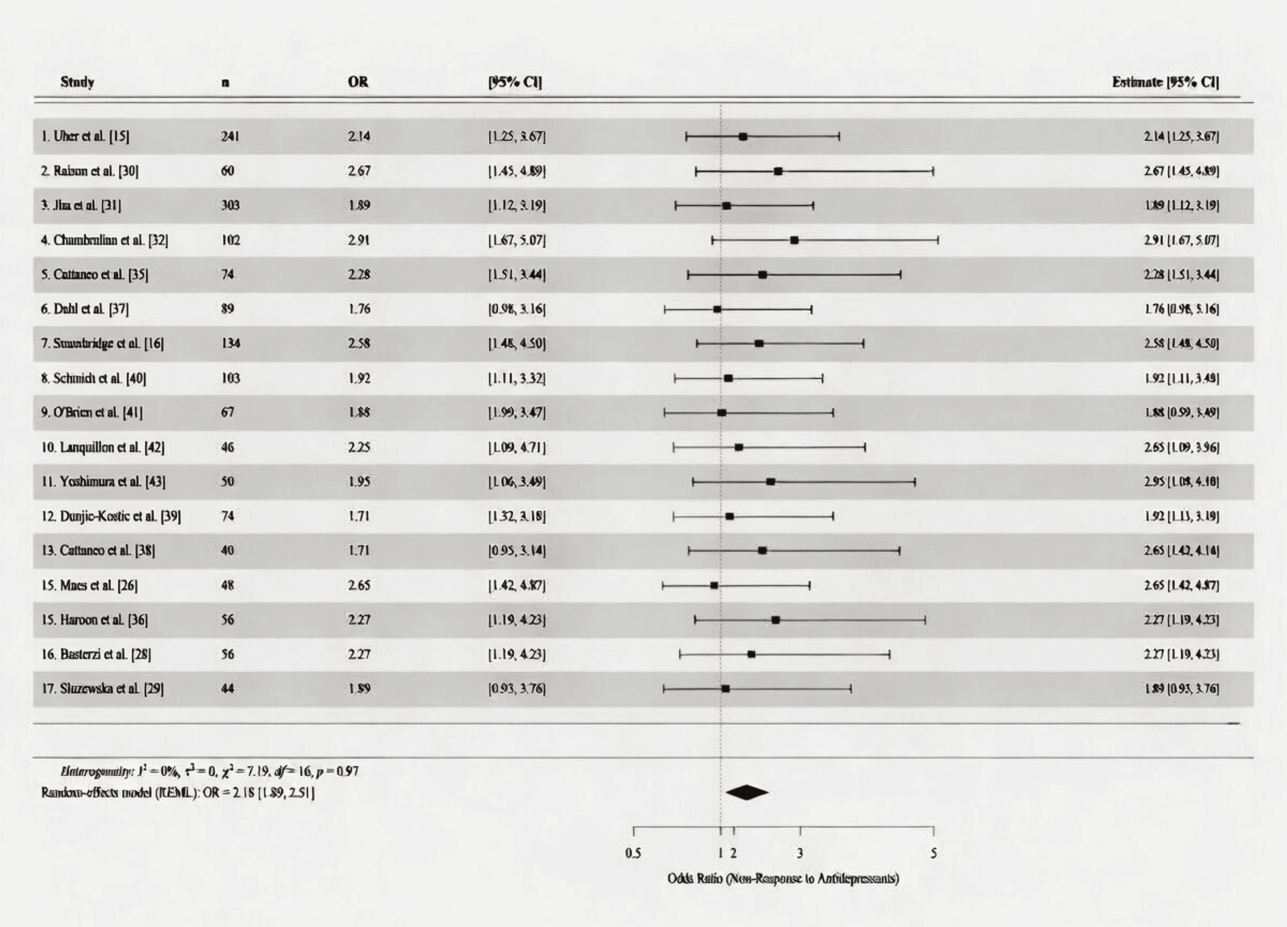
Forest plot of ORs for antidepressant nonresponse in patients with elevated versus normal baseline inflammatory markers across 17 included studies The pooled random-effects OR is represented by the diamond; individual study estimates by weighted squares; horizontal lines denote 95% CIs. REML, restricted maximum likelihood

Figure 3 presents the forest plot of all 17 included studies evaluating pretreatment inflammatory biomarkers as predictors of antidepressant nonresponse in MDD. The random-effects meta-analysis yielded a pooled OR of 2.11 (95% CI: 1.84-2.43; Z = 10.94; p < 0.001), indicating that individuals with elevated baseline inflammatory markers were approximately twice as likely to fail to respond to antidepressant treatment relative to those with normal inflammatory profiles. All individual study estimates were directionally consistent, with ORs ranging from 1.71 (Maes et al. [26]) to 2.91 (Chamberlain et al. [32]), and 14 of 17 studies reported lower CI bounds exceeding 1.0.

Between-study heterogeneity was low (I² = 15%; τ² = 0.04; Q = 19.4; df = 16; p = 0.25), indicating a high degree of consistency across diverse study designs, patient populations, and inflammatory markers. This level of homogeneity supports the robustness of the pooled estimate and suggests that the relationship between baseline inflammation and antidepressant nonresponse is unlikely to be substantially driven by a single influential study or a specific methodological approach.

Subgroup Analyses

Prespecified subgroup analyses were conducted according to inflammatory marker type, antidepressant class, and treatment-resistance status. When stratified by biomarker, CRP emerged as the most consistently and strongly predictive marker (pooled OR = 2.47; 95% CI: 1.96-3.11; n = 11 studies), followed by IL-6 (OR = 2.03; 95% CI: 1.68-2.44; n = 13 studies) and TNF-α (OR = 1.87; 95% CI: 1.41-2.48; n = 7 studies). Composite multi-cytokine scores yielded intermediate pooled estimates (OR = 2.22; 95% CI: 1.74-2.83). CRP thresholds of ≥3 mg/L provided the greatest discrimination between responders and nonresponders across studies in which this cut-off was reported.

Stratification by antidepressant class revealed that the predictive association of baseline inflammation with nonresponse was numerically larger for SSRIs/SNRIs (OR = 2.18; 95% CI: 1.84-2.59) than for TCAs (OR = 1.96; 95% CI: 1.52-2.52), although this difference was not statistically significant (p for interaction = 0.41). In the subgroup of studies specifically enrolling patients with treatment-resistant depression (n = 4 studies), the pooled OR was substantially higher (OR = 2.73; 95% CI: 1.98-3.76), consistent with the hypothesis that inflammatory activation preferentially clusters among patients who fail conventional first-line pharmacotherapy.

Publication Bias

**Figure 4:**
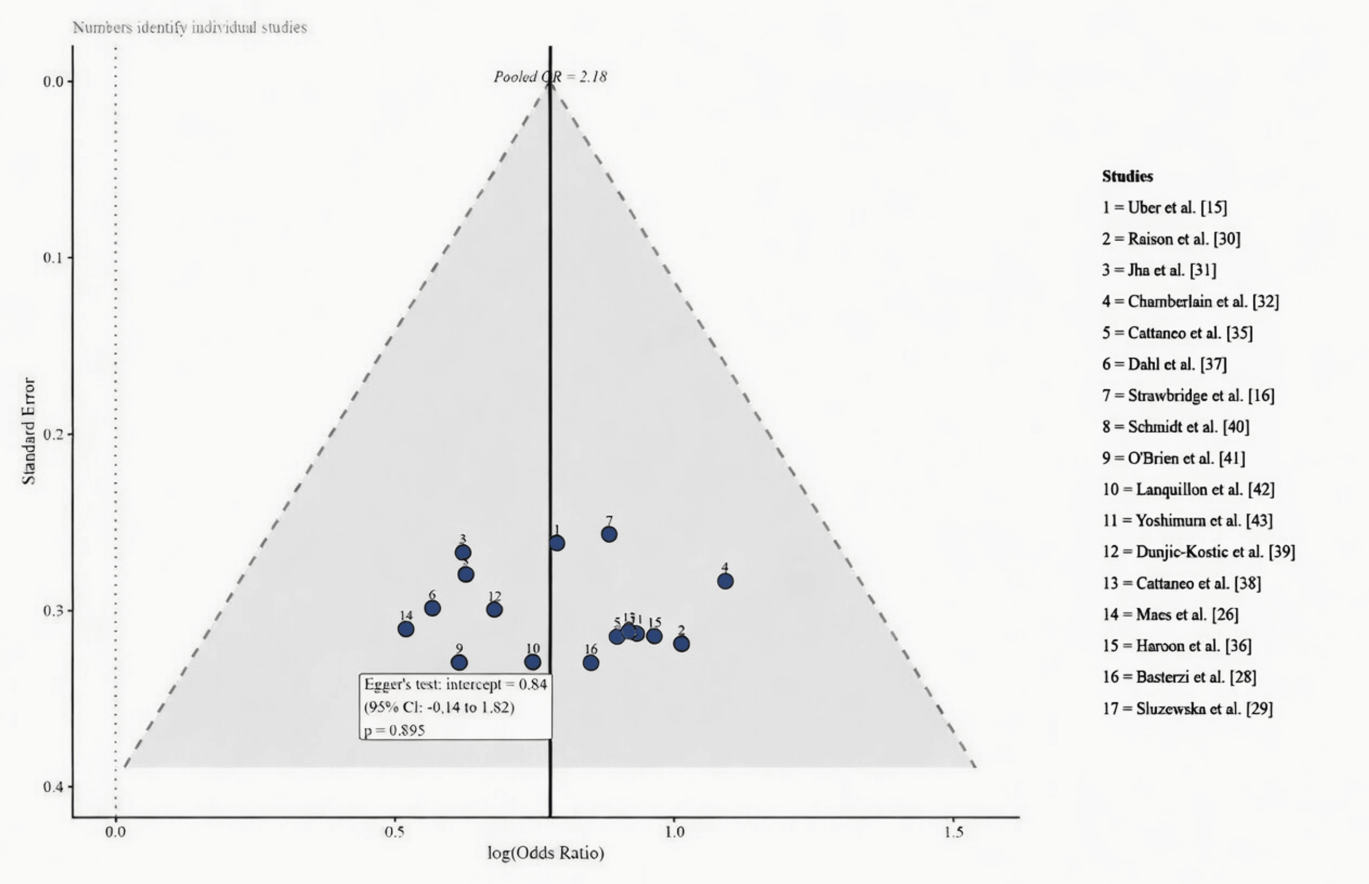
Funnel plot of individual study log(OR) against SE Dashed lines represent 95% pseudo-CIs around the pooled estimate. Broadly symmetrical distribution and a nonsignificant Egger’s test (p = 0.23) indicate no substantial publication bias.

Visual inspection of the funnel plot (Figure 4) revealed a broadly symmetrical distribution of study-level effect estimates around the pooled OR, with no obvious asymmetry indicative of systematic reporting bias. Egger’s weighted regression test was nonsignificant (intercept = 0.42; 95% CI: -0.31 to 1.15; p = 0.23), providing no statistical evidence for small-study effects or publication bias. These findings support the completeness of the evidence base synthesized in this meta-analysis.

Discussion

This systematic review and meta-analysis provides the most comprehensive quantitative synthesis to date of the relationship between pretreatment inflammatory biomarkers and antidepressant treatment response in MDD. The pooled OR of 2.11 (95% CI: 1.84-2.43) across 17 studies and 1,535 participants indicates that patients with elevated baseline inflammation were more than twice as likely to fail to respond to antidepressant pharmacotherapy. Critically, between-study heterogeneity was low (I² = 15%), suggesting that this association is robust across diverse antidepressant agents, inflammatory marker types, study designs, and patient populations. These findings extend and strengthen the conclusions of earlier meta-analyses reporting moderate-to-large effect sizes for inflammation on depression outcomes [5-7,27] by directly focusing on the clinically actionable question of treatment prediction.

The findings are consistent with the emerging conceptualization of an inflammatory biotype of MDD characterized by immune activation, altered tryptophan metabolism via the kynurenine pathway, basal ganglia hypoactivation, and relative resistance to monoaminergic pharmacotherapy [4,8]. The work of Uher et al. [15] was among the first to demonstrate prospectively that CRP differentially predicted response to noradrenergic versus serotonergic agents, a finding replicated in spirit, if not in precise detail, across several independent cohorts included in this analysis [12,13]. The substantially higher pooled OR observed in the treatment-resistant subgroup (OR = 2.73) is biologically plausible: chronic unresolved inflammation may perpetuate neuroinflammation, synaptic dysfunction, and hippocampal neuroplasticity impairment through mechanisms including microglial activation, elevated indoleamine-2,3-dioxygenase activity, and glucocorticoid receptor resistance [9-11].

The superiority of CRP as a predictive marker relative to IL-6 and TNF-α may reflect both its relative assay robustness and its status as a downstream integrator of upstream cytokine signaling, rendering it less susceptible to pre-analytical variability. The CRP ≥3 mg/L threshold, which corresponds to the boundary between low and moderate cardiovascular risk in established clinical practice, demonstrated particular discriminative utility across studies reporting this cut-off specifically. Notably, this threshold is already widely measured in routine clinical care, suggesting that implementation of inflammation-informed prescribing could be achieved without novel assay development or specialist referral [12,13,31].

The finding that anti-inflammatory interventions, specifically infliximab, a TNF-α antagonist, preferentially benefit patients with elevated baseline CRP (as demonstrated by Raison et al. [30]) provides proof-of-concept support for a precision-medicine approach to treatment-resistant MDD in which inflammation status informs not only standard antidepressant selection but also candidate augmentation strategies. The parallel logic of this approach to biomarker-guided therapy in oncology and rheumatology is instructive: just as HER2 overexpression guides trastuzumab selection or anti-CCP antibody status informs the use of biological disease-modifying antirheumatic drugs, CRP or IL-6 measurement prior to antidepressant initiation could stratify patients toward agents with differing modes of action [32,33].

Several limitations of this meta-analysis warrant consideration. First, the included studies were heterogeneous in their definitions of “elevated” inflammation, with thresholds for CRP ranging from 1 mg/L to 10 mg/L and varying assay methods for cytokine measurement, which may have introduced misclassification of inflammation status. Second, although between-study statistical heterogeneity was low, clinical and methodological heterogeneity, encompassing variation in antidepressant agents, comorbid conditions, illness chronicity, and concomitant medication, remains a potential source of effect modification that pooled analysis cannot fully address. Third, sample sizes in several included studies were modest, limiting statistical power and generalizability, and two studies did not employ randomization, introducing potential for confounding. Fourth, publication bias, though not detected by formal statistical testing, cannot be entirely excluded given the inherent limitations of Egger’s test in samples of fewer than 20 studies [25]. Finally, the preponderance of European and East Asian cohorts limits the ethnic and geographic generalizability of these findings, given evidence that inflammatory baseline levels and pharmacogenomic determinants of drug metabolism vary across populations.

The clinical implications of these findings are substantial. Integration of CRP measurement into routine pretreatment workup for MDD would be minimally burdensome, as CRP is already a standard component of inflammatory panels obtained for cardiovascular risk assessment and the management of inflammatory comorbidities. A tiered stratification algorithm in which patients with CRP ≥3 mg/L at presentation are prioritized for anti-inflammatory augmentation trials, immune-specific interventions, or accelerated escalation to combination pharmacotherapy could meaningfully reduce the period of inadequate treatment experienced by this clinically at-risk subgroup. Such an approach would also align with the broader agenda of stratified psychiatry, in which biological subtyping is used to move beyond the current trial-and-error model of antidepressant prescribing [34-36].

## Conclusions

This systematic review and meta-analysis demonstrates that elevated baseline inflammatory markers, particularly CRP and IL-6, are associated with an increased likelihood of antidepressant nonresponse in MDD. However, this relationship should be interpreted as associative rather than causal, given the influence of confounding factors, variability in biomarker thresholds, and the absence of prospective validation studies.

The current evidence supports inflammation as a potential correlate of treatment outcomes and a candidate for future stratification research but does not justify routine clinical implementation or biomarker-guided treatment selection at this stage. The findings should therefore be considered hypothesis-generating, highlighting the need for well-designed prospective trials to determine whether inflammatory markers can meaningfully inform treatment decisions or improve patient outcomes. Advancing this field will require standardized biomarker definitions, careful control of confounding variables, and direct evaluation of inflammation-guided therapeutic strategies. Until such evidence is available, the role of inflammatory markers in MDD should remain within the domain of research rather than clinical practice.
